# Supplementary material for: Macropinocytosis drives T cell growth by sustaining the activation of mTORC1
Source: Nat Commun. 2020 Jan 10;11:180. doi: 10.1038/s41467-019-13997-3 (PMC6954116; doi:10.1038/s41467-019-13997-3)
Supplement: Supplementary file 1 — Supplementary Information [file 41467_2019_13997_MOESM1_ESM.pdf]

Supplementary Information

Macropinocytosis drives T cell growth by sustaining the activation of mTORC1

John C. Charpentier, Di Chen, Philip E. Lapinski, Jackson Turner, Irina Grigorova, Joel A. Swanson & Philip D. King

Includes Supplementary Figures 1-8

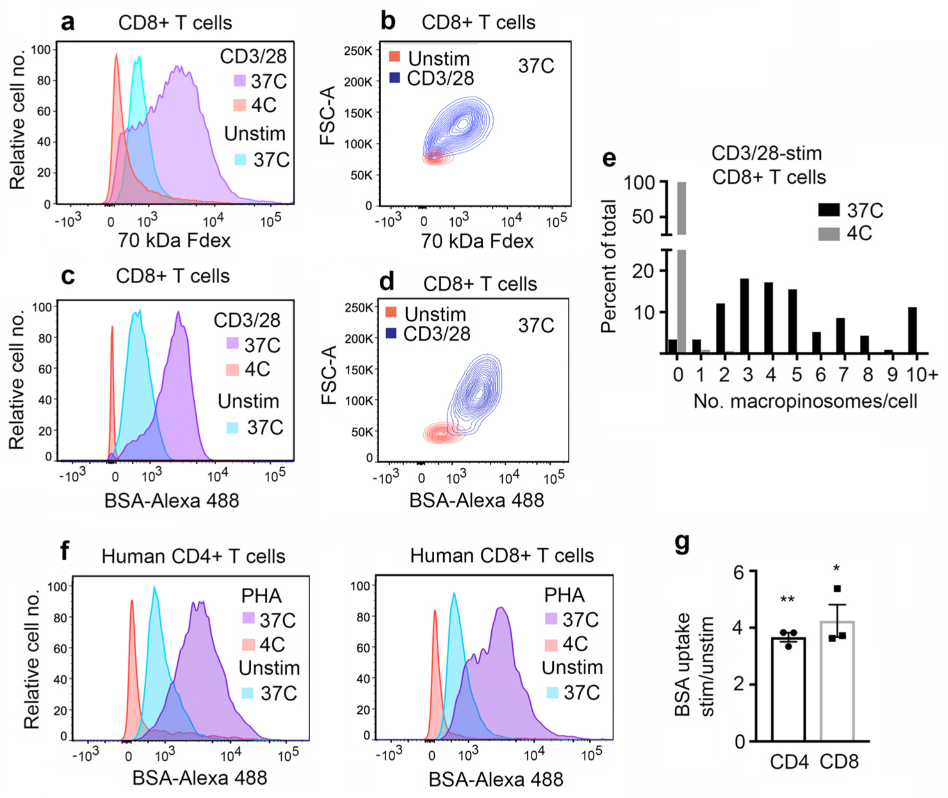

Supplementary Figure 1. T cell uptake of macropinocytosis probes. a-d, Murine splenocytes were unstimulated or stimulated with CD3/28 mAb for 24 h (**a,b**) or 20 h (**c,d**). 70 kDa Fdex (**a,b**) or BSA-Alexa 488 (**c,d)** probes were incubated with cells for the last 4 h or 8 h of culture respectively at the indicated temperatures. Representative flow cytometry histogram plots of CD8+ T cell probe uptake and contour plots of probe uptake versus FSC-A are shown (a,b, n=10; c,d, n=12 independent experiments). **e,** Quantitation of the number of macropinosomes per CD8+ T cell stimulated with CD3/28 mAb as in Fig. 1h (n=116 cells at 37^0C and 4^0C). **f,g**, Human PBMC were unstimulated or stimulated with PHA for 20 h. Cells were incubated with BSA-Alexa 488 for the last 8 h of culture at the indicated temperatures. **f**, Representative flow cytometry histogram plots of CD4+ and CD8+ T cell probe uptake. **g**, Mean +/- 1 SEM of the ratio of BSA probe uptake in stimulated versus unstimulated CD4+ and CD8+ T cells at 37^0C (n=3 independent experiments). **P*<0.05, ***P*<0.01 by Student's 1-sample 2-sided *t*-test. Source data are provided as Source Data file.

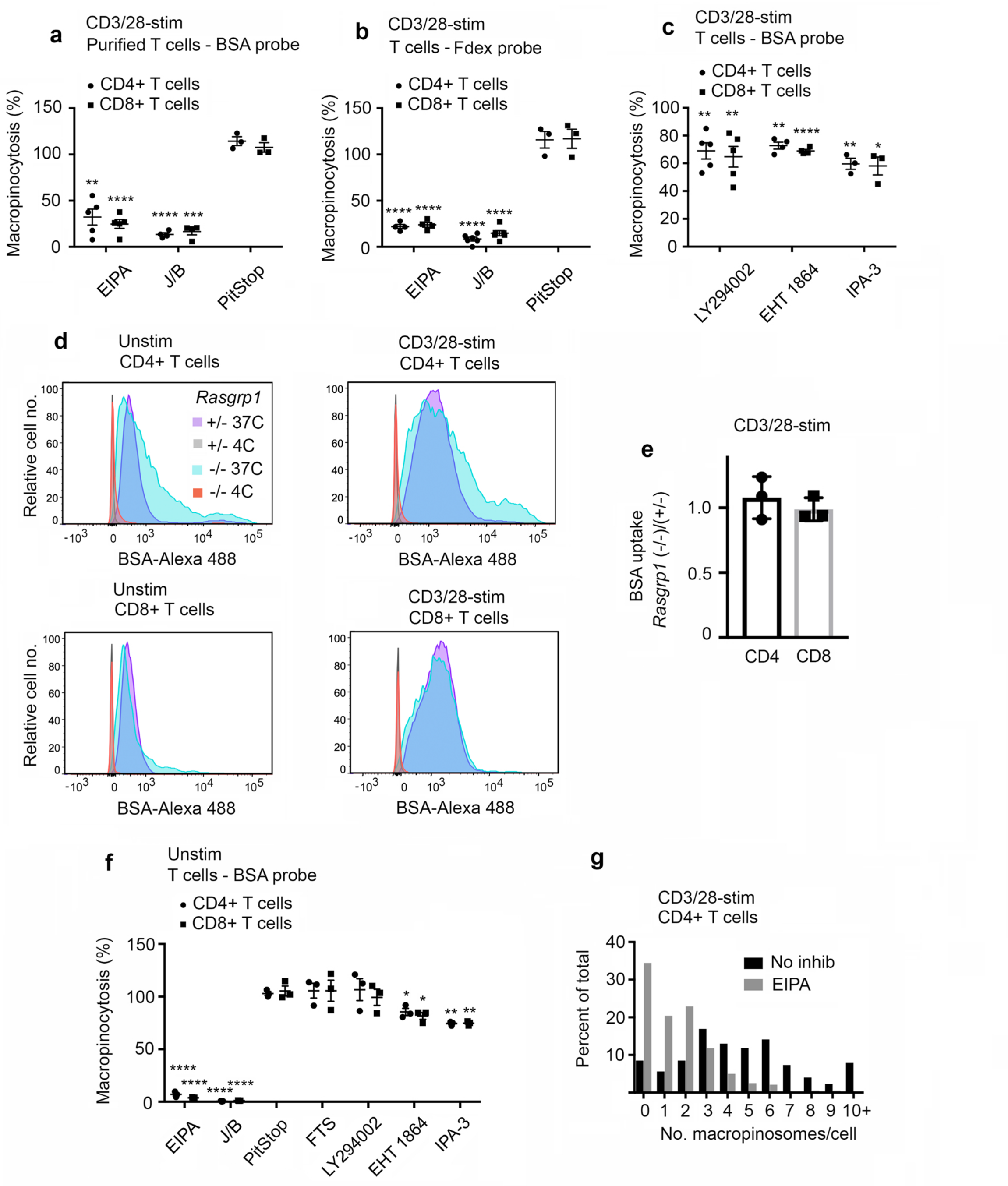

Supplementary Figure 2. Inhibitors of macropinocytosis block T cell uptake of BSA. a-f, Purified pan T cells (**a**) or splenocytes (**b-f**) from wild-type (**a-c, f**) or *Rasgrp1* mutant (**d,e**) mice were unstimulated or stimulated with CD3/28 mAb for 12 h before incubation with BSA-Alexa 488 (**a,c-f**) or 70 kDa Fdex (**b**) at 37^{0}C or 4^{0}C in the presence or absence of the indicated inhibitors for a further 2 h (**f**) or 8 h (**a-e**). **a-c,f**, Mean +/- 1 SEM of the percentage macropinocytosis relative to the positive control. **a,** (EIPA, n=5; J/B, n=4; PitStop, n=3 independent experiments). **b**, (EIPA, n=4; J/B, n=6; PitStop, n=3). **c**, (LY294002, n=5; EHT 1864, n=4; IPA-3, n=3 independent experiments). **f**, n=3 independent experiments for each inhibitors. **P*<0.05, ***P*<0.01, ****P*<0.001, *****P*<0.0001 by Student's 1-sample 2-sided *t*-test. **d,e,** Representative flow cytometry histogram plots (**d**) and mean +/- 1 SEM of the ratio of probe uptake by stimulated *Rasgrp1* +/- and -/- CD4+ and CD8+ T cells (**e**) (n=3 independent experiments). **g**, Quantitation of the number of macropinosomes per CD4+ T cell stimulated with CD3/28 mAb as in Fig. 3h (n=177 and 279 cells respectively) . Source data are provided as Source Data file.

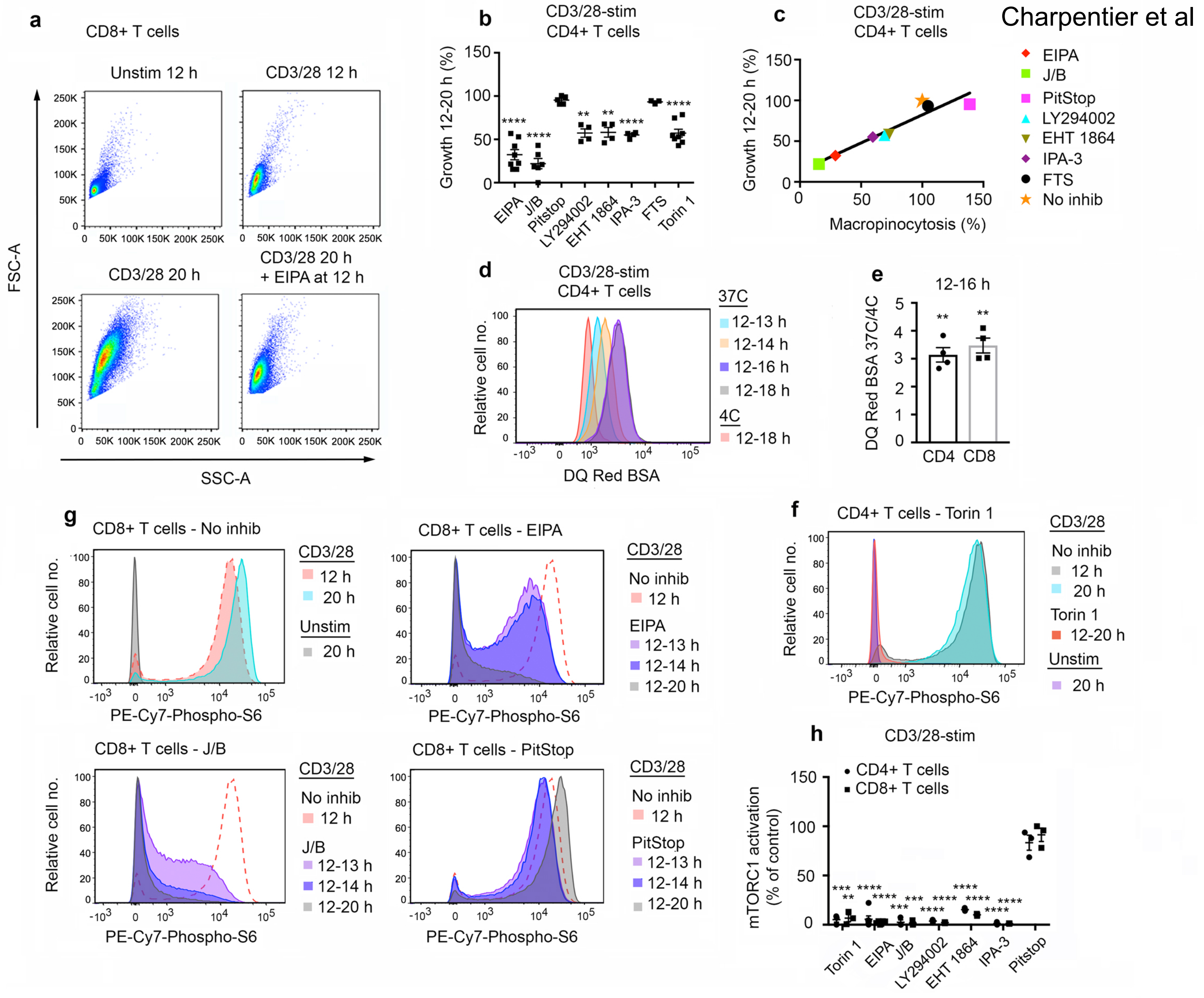

Supplementary Figure 3. T cell growth and activation of mTORC1 is dependent upon macropinocytosis. **a-c**, Splenocytes were unstimulated or stimulated with CD3/28 mAb for 12 h or were stimulated with CD3/28 mAb for 20 h in the absence or presence of the indicated inhibitors that were added to cultures at the 12 h time point. **a,** Shown are flow cytometry scatter plots of FSC-A versus SSC-A for CD8+ T cells from a single experiment (representative of 8 performed with EIPA). **b**, For each inhibitor, the mean percentage +/- 1 SEM of CD4+ T cell growth between 12 and 20 h relative to growth in the absence of inhibitor in the same experiment is shown (see Methods) (EIPA, n=8; J/B, n=6; PitStop, n=5; LY294002, n=4; EHT 1864, n=4; IPA-3, n=4; FTS, n=3; Torin 1, n=8 independent experiments). ***P*<0.01, *****P*<0.0001 by Student's 1-sample 2-sided *t*-test. **c**, Graph shows mean percentage macropinocytosis (Fig. 3b and Supplementary Fig. 2c) versus mean percentage growth (b) for CD4+ T cells for each inhibitor. **d,e**, Splenocytes were stimulated with CD3/28 mAb for 12 h before incubation with DQ Red BSA for the indicated times at the indicated temperatures. Shown is a representative flow cytometry histogram plot of DQ Red BSA fluoresence in CD4+ T cells (**d**) and mean +/- 1 SEM of the ratio of DQ Red BSA fluoresence in CD4+ and CD8+ T cells at 37^{0}C versus 4^{0}C after probe incubation between 12 and 16 h (**e**) (n=4 independent experiments). ***P*<0.01 by Student's 1-sample 2-sided *t*-test. **f,g,** Splenocytes were unstimulated or stimulated with CD3/28 mAb for different times in the absence or presence of inhibitors added at 12 h. Flow cytometry histograms show relative amounts of phospho-S6 in CD4+ T cells (**f**) or CD8+ T cells (**g**). All panels in (**g**) are from the same experiment. Plots are representative of multiple repeat experiments (n=6 for EIPA and n=3 independent experiments for Torin 1, J/B and PitStop). **h**, Mean +/- 1 SEM of the percentage of mTORC1 activation at 20 h relative to the positive control following addition of inhibitors at 12 h calculated as indicated in Methods (n=6 for EIPA and n=3 independent experiments for all other inhibitors). ***P*<0.01, ****P*<0.001, *****P*<0.0001 by Student's 1-sample 2-sided *t*-test. Source data are provided as Source Data file.

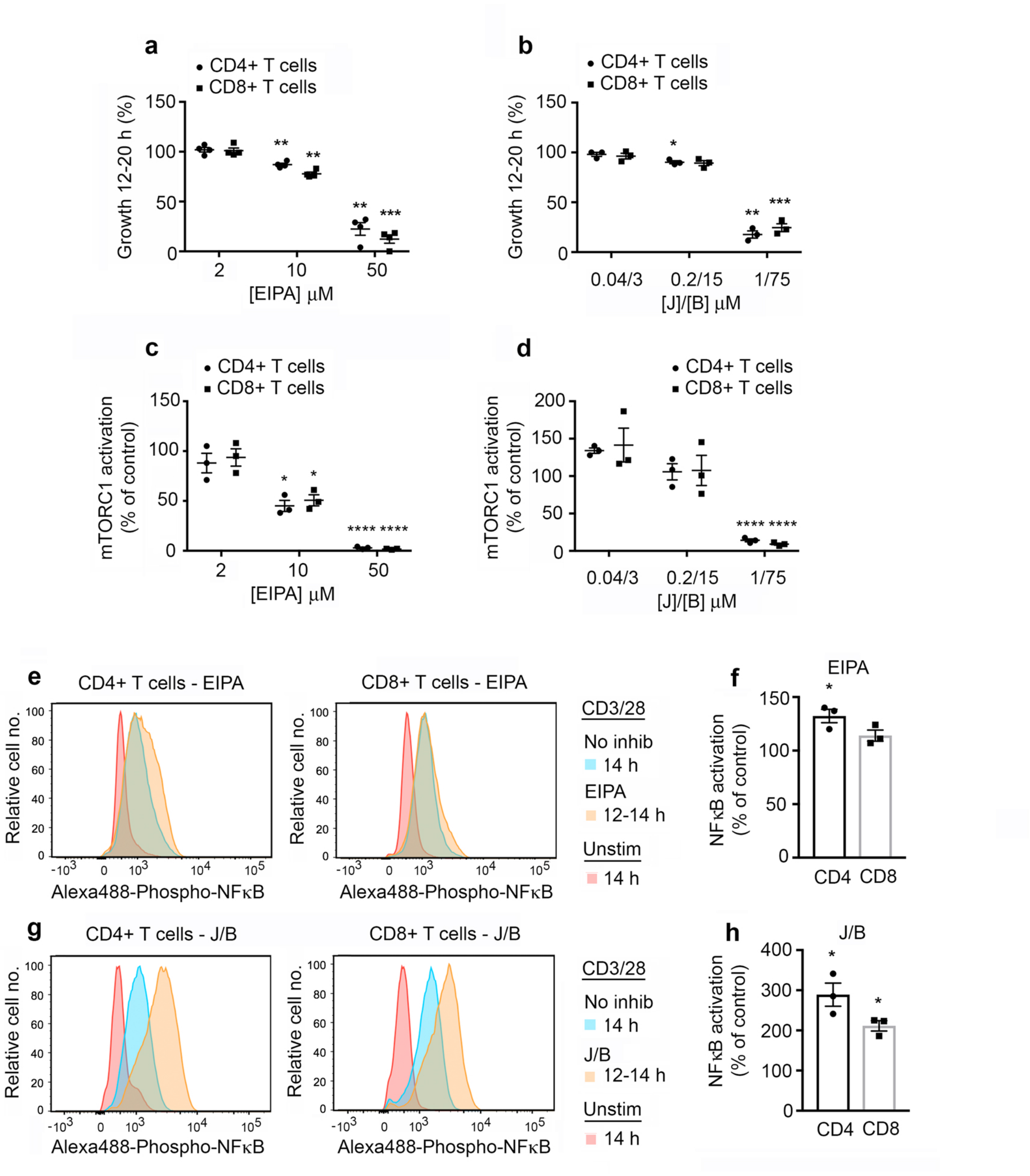

Supplementary Figure 4. Role of macropinocytosis in T cell growth and mTORC1 and NFκB activation. **a-h,** Murine splenocytes were stimulated with CD3/28 mAb for 20 h (**a-d**) or 14 h (**e-h**) in the absence or presence of the indicated inhibitors that were added to cultures at 12 h. In **e-h**, the highest concentrations of inhibitors shown in **a-d** were used. **a-d,** Mean percentage +/- 1 SEM of T cell growth between 12 and 20 h (**a,b**) and the percentage of mTORC1 activation at 20 h (**c,d**) (see Methods). **a,** n=4. **b-d,** n=3 independent experiments. **P*<0.05, ***P*<0.01, ****P*<0.001, *****P*<0.0001 by Student's 1-sample 2-sided *t*-test. **e-h**, Relative amounts of phospho-NFκB in CD4+ and CD8+ T cells at 14 h were determined by flow cytometry. **e,g,** Representative flow histograms showing effect of EIPA and J/B upon phospho-NFκB. **f,h,** Mean +/- 1 SEM of the percentage of NFκB activation at 14 h relative to the positive control (see Methods). n=3 independent experiments. **P*<0.05, by Student's 1-sample 2-sided *t*-test. Source data are provided as Source Data file.

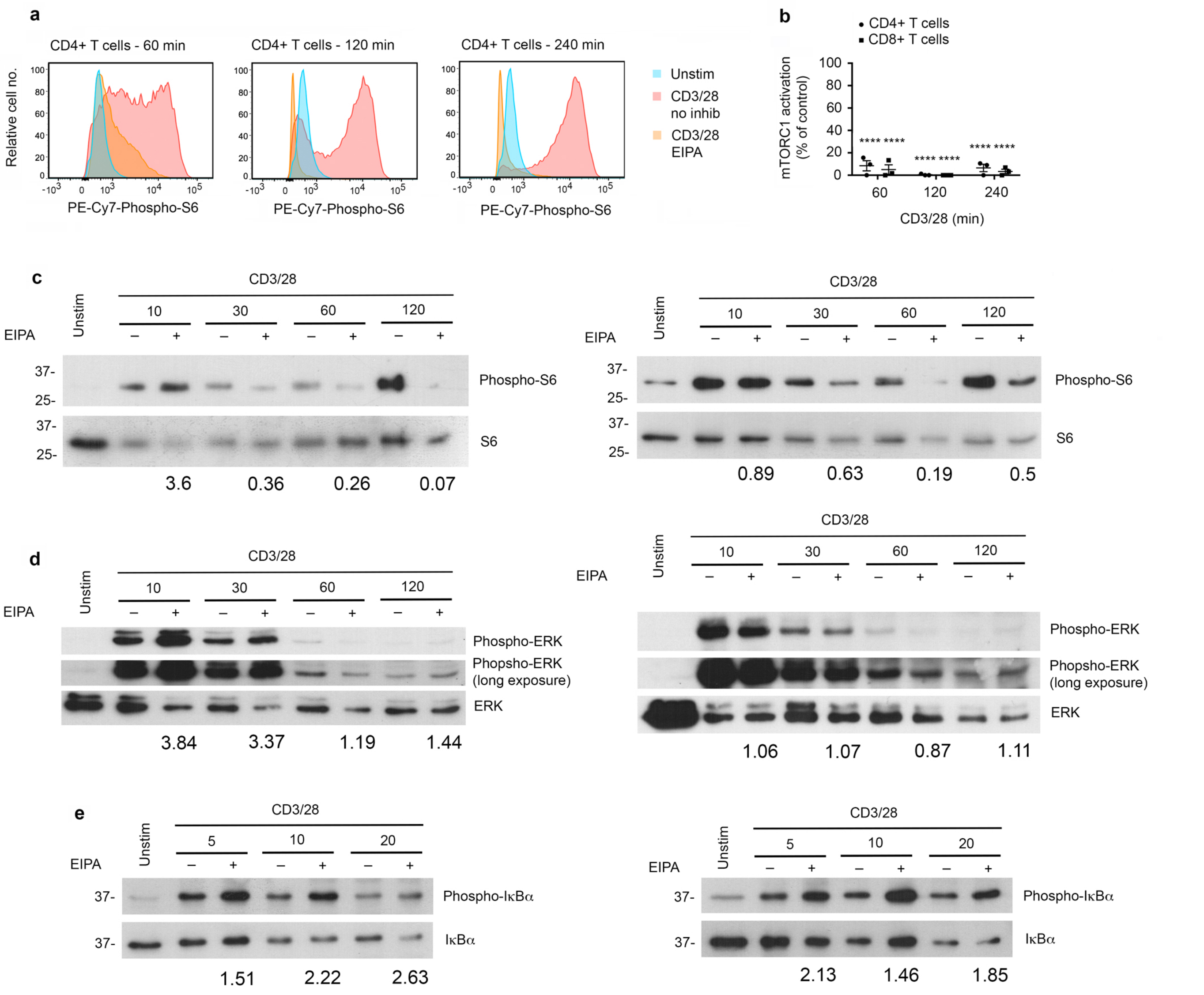

Supplementary Figure 5. Specific inhibition of mTORC1 by EIPA in acutely activated T cells. a-e, Splenocytes (**a,b**) or purified splenic CD4+ T cells (**c-e**) were stimulated with CD3/28 mAb for the indicated times (in min) in the presence or absence of EIPA added at culture initiation. **a,b,** Relative amounts of phospho-S6 in CD4+ and CD8+ T cells were determined by flow cytometry. Shown are flow cytometry plots for CD4+ T cells from a representative experiment (**a**) and mean +/- 1 SEM of the percentage of mTORC1 activation at each time point for CD4+ and CD8+ T cells (n=3 independent experiments) (see Methods) (**b**). *****P*<0.0001 by Student's 1-sample 2-sided *t*-test. **c-e,** Activation of mTORC1 (**c**), ERK MAPK (**d**), and NFκB (**e**) was determined by Western blotting using phospho-specific-S6, -ERK and -IκBα antibodies respectively. The magnitude of phospho-signals was normalized to the amounts of corresponding non-phosphorylated species following blot stripping and re-probing with specific antibodies. Numbers below EIPA tracks represent the ratio of normalized phospho signals of EIPA-treated to non-treated T cells at each time point of CD3/28 mAb stimulation. Shown are repeat experiments for each molecular species. Source data are provided as Source Data file.

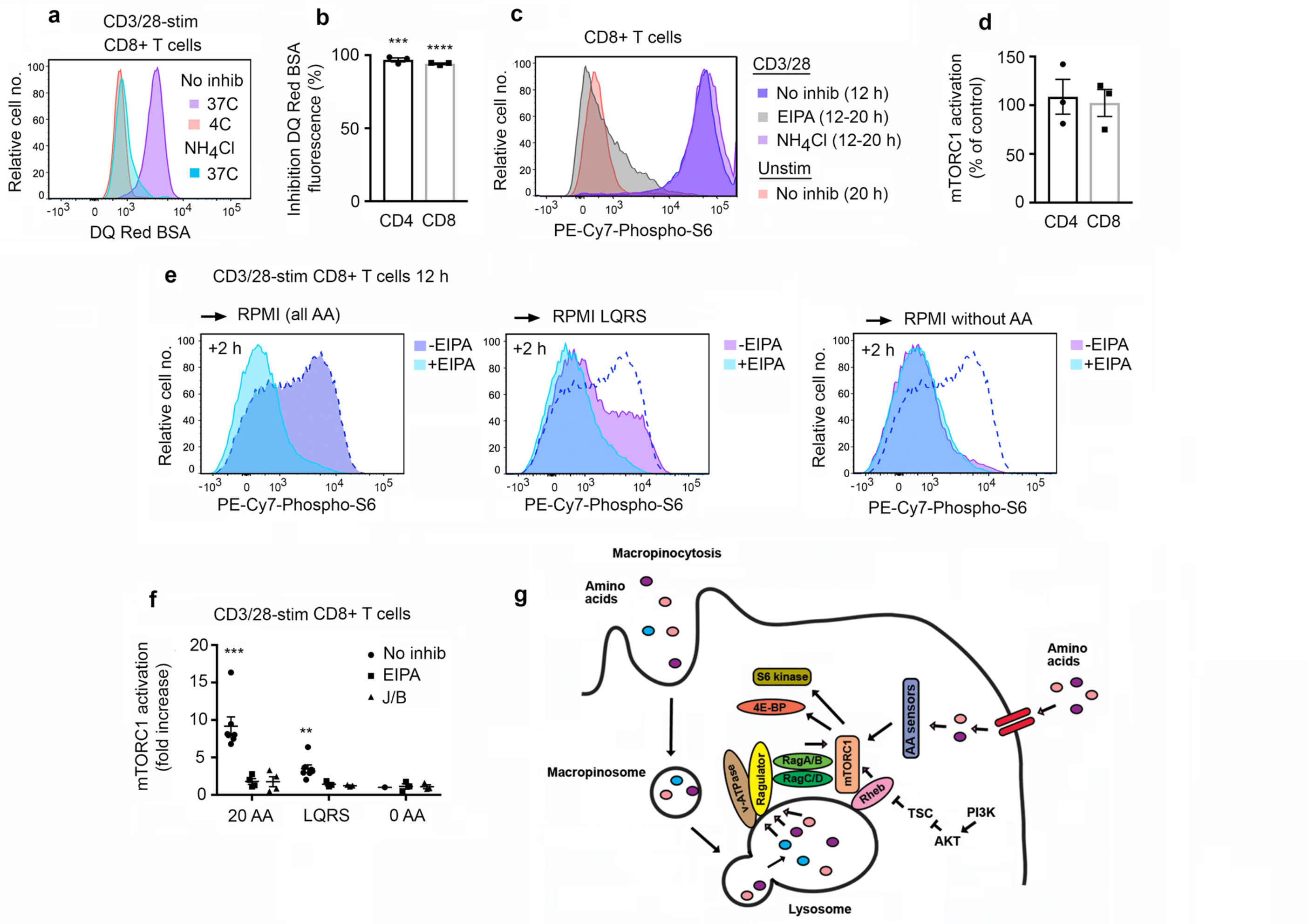

Supplementary Figure 6. Macropinocytosis of free AA sustains mTORC1 activation in T cells. a-d, Splenocytes were stimulated with CD3/28 mAb for 12 h followed by incubation with DQ Red BSA for 4 h at the indicated temperatures in the presence or absence of NH_4Cl (**a,b**) or for 8 h at 37^0C in the presence or absence of EIPA or NH_4Cl (**c,d**). **a,c,** Representative flow cytometry histogram plots of DQ Red BSA fluorescence (**a**) or phospho-S6 levels (**c**) in CD8+ T cells. **b**, Mean percentage +/- 1 SEM of inhibition of DQ Red BSA fluorescence in CD4+ and CD8+ T cells in the presence of NH_4Cl calculated as described in Methods (n=3 independent experiments). **d**, Mean +/- 1 SEM of the percentage of mTORC1 activation at 20 h relative to the positive control following addition of NH_4Cl at 12 h calculated as indicated in Methods (n=3 independent experiments). ****P*<0.001, *****P*<0.0001 by Student's 1-sample 2-sided *t*-test. **e**, Splenocytes were stimulated with CD3/28 mAb in complete medium (RPMI plus FCS) for 12 h, washed and re-cultured in the indicated media for 2h in the presence or absence of EIPA. Representative flow cytometry histograms show phospho-S6 levels in CD4+ T cells. All panels are from the same experiment. The mid-blue-dashed line indicates all AA in the absence of EIPA. **f**, Mean +/- 1 SEM of the fold increase in mTORC1 activation in CD8+ T cells at 14 h relative to the 0 AA control in the absence of inhibitors (n=7 independent experiments for 20 AA and LQRS in the absence of inhibitors; n=4 independent experiments for 20 AA and n=3 independent experiments for LQRS and 0 AA in the presence of inhibitors). ***P*<0.01, ****P*<0.001 by Student's 1-sample 2-sided *t*-test. **g**, Model of mTORC1 activation in stimulated T cells. Macropinocytosis delivers free AA from the extracellular space to lysosomes in T cells where they modulate the activity of the Ragulator complex resulting in recruitment of mTORC1 to the lysosomal membrane. PI3K signals emanating from cell surface receptors lead to activation of the Rheb small GTPase on lysosomes, which, in turn, activates localized mTORC1. mTORC1 phosphorylates p70 S6 kinase and 4E-BP that promote anabolic processes and T cell growth. AA transporters in T cells permit entry of AA into the cytosol where they are detected by cytosolic AA sensors that provide additional necessary signals for mTORC1 activation. Source data are provided as Source Data file.

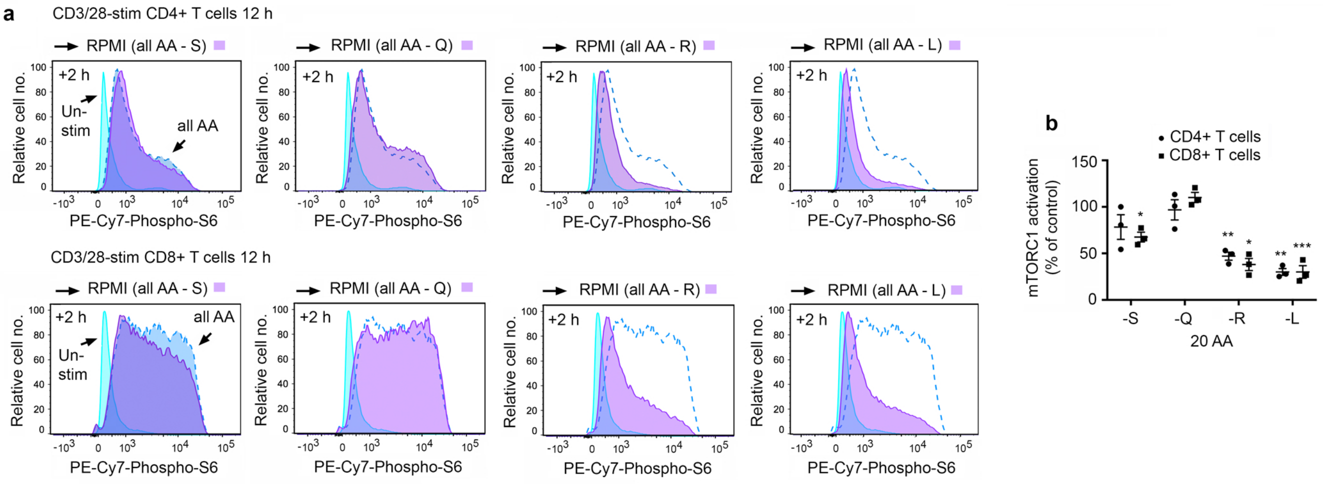

Supplementary Figure 7. Role of leucine and arginine in sustained activation of mTORC1 in T cells. a,b, Splenocytes were stimulated with CD3/28 mAb in complete medium (RPMI plus FCS) for 12 h, washed and re-cultured in the indicated media for 2h. **a,** Representative flow cytometry histograms show phospho-S6 levels in CD4+ and CD8+ T cells. All panels are from the same experiment. The mid-blue-dashed line indicates all AA. The light blue histogram indicates negative control unstimulated T cells at 12 h. **b,** Mean +/- 1 SEM of the percentage mTORC1 activation in CD4+ and CD8+ T cells at 14 h relative to the all 20 AA control (n=3 independent experiments). **P*<0.05, ***P*<0.01, ****P*<0.001 by Student's 1-sample 2-sided *t*-test. Source data are provided as Source Data file.

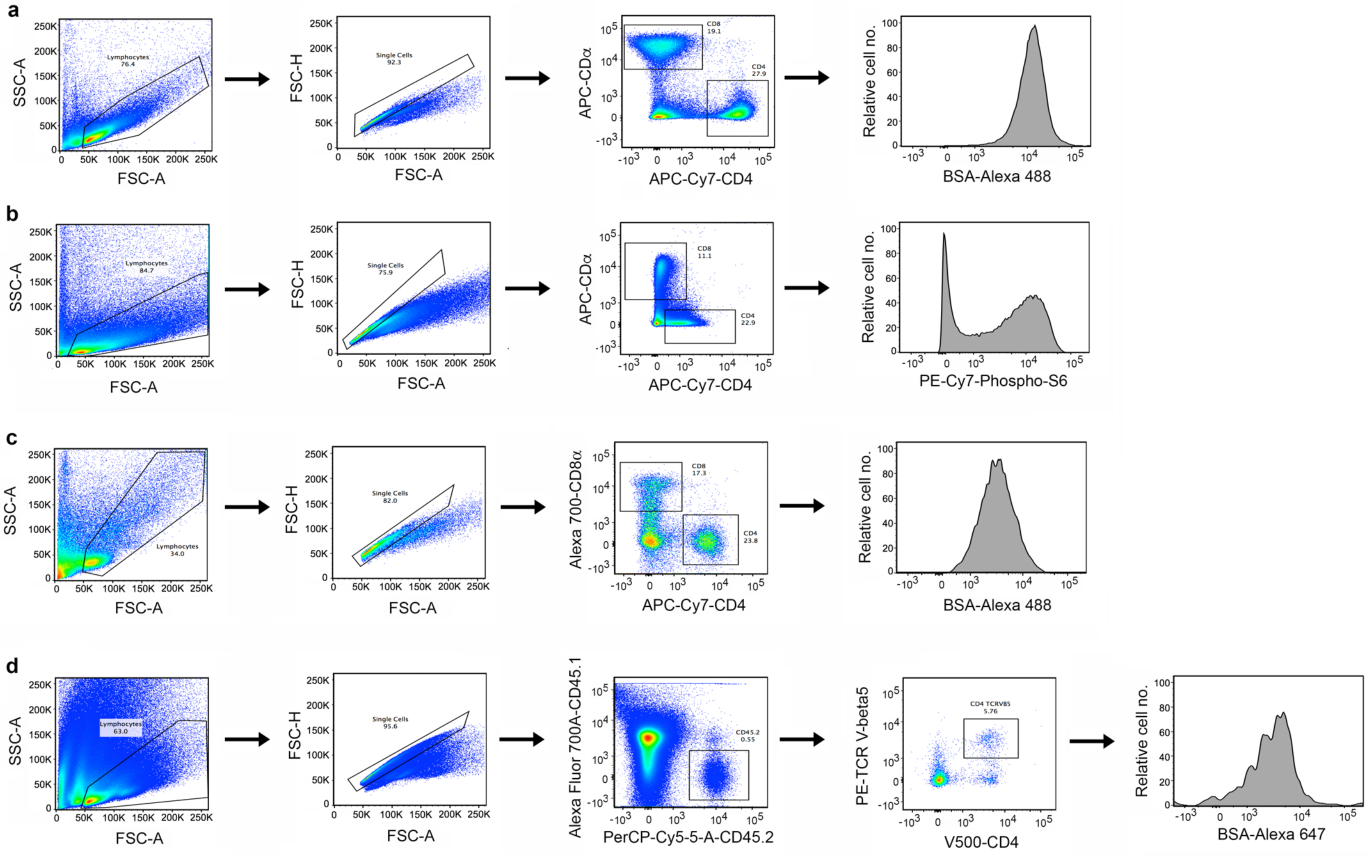

Supplementary Figure 8. Flow cytometry gating strategies. a, Gating strategies used to determine: **a**, T cell uptake of macropinocytosis probes in murine splenocyte cultures; **b**, Phospho-S6 or phospho-NFκB staining in fixed and permeabilized T cells in murine splenocyte cultures; **c**, T cell uptake of macropinocytosis probes in human PBMC cultures; **d**, OTII TCR Tg T cell uptake of macropinocytosis probes in vivo.
