# Supplementary material for: Macropinocytosis drives T cell growth by sustaining the activation of mTORC1
Source: Nat Commun. 2020 Jan 10;11:180. doi: 10.1038/s41467-019-13997-3 (PMC6954116; doi:10.1038/s41467-019-13997-3)
Supplement: Supplementary file 3 — Reporting Summary [file 41467_2019_13997_MOESM3_ESM.pdf]

nature research

Corresponding author(s): Philip D. King

Last updated by author(s): Dec 9, 2019

Reporting Summary

Nature Research wishes to improve the reproducibility of the work that we publish. This form provides structure for consistency and transparency in reporting. For further information on Nature Research policies, see Authors & Referees and the Editorial Policy Checklist.

Statistics

For all statistical analyses, confirm that the following items are present in the figure legend, table legend, main text, or Methods section.

n/a	Confirmed	
☐	☒	The exact sample size (n) for each experimental group/condition, given as a discrete number and unit of measurement
☐	☒	A statement on whether measurements were taken from distinct samples or whether the same sample was measured repeatedly
☐	☒	The statistical test(s) used AND whether they are one- or two-sided<br>*Only common tests should be described solely by name; describe more complex techniques in the Methods section.*
☐	☒	A description of all covariates tested
☐	☒	A description of any assumptions or corrections, such as tests of normality and adjustment for multiple comparisons
☐	☒	A full description of the statistical parameters including central tendency (e.g. means) or other basic estimates (e.g. regression coefficient) AND variation (e.g. standard deviation) or associated estimates of uncertainty (e.g. confidence intervals)
☐	☒	For null hypothesis testing, the test statistic (e.g. F, t, r) with confidence intervals, effect sizes, degrees of freedom and P value noted<br>*Give P values as exact values whenever suitable.*
☒	☐	For Bayesian analysis, information on the choice of priors and Markov chain Monte Carlo settings
☒	☐	For hierarchical and complex designs, identification of the appropriate level for tests and full reporting of outcomes
☒	☐	Estimates of effect sizes (e.g. Cohen's d, Pearson's r), indicating how they were calculated

Our web collection on statistics for biologists contains articles on many of the points above.

Software and code

Policy information about availability of computer code

Data collection	Images were acquired using Leica LAS AF software; Flow cytometry data were acquired using BD FACSDiva v. 8.01.
Data analysis	Flow cytometry data were analyzed in Flowjo v. 10.1; Image deconvolution was performed in Huygens Professional v. 17.04.0p5; Statistical analyses were performed using GraphPad Prism v. 7.

For manuscripts utilizing custom algorithms or software that are central to the research but not yet described in published literature, software must be made available to editors/reviewers. We strongly encourage code deposition in a community repository (e.g. GitHub). See the Nature Research guidelines for submitting code & software for further information.

Data

Policy information about availability of data

All manuscripts must include a data availability statement. This statement should provide the following information, where applicable:

- Accession codes, unique identifiers, or web links for publicly available datasets
- A list of figures that have associated raw data
- A description of any restrictions on data availability

The flow cytometry and imaging data supporting these findings are available from the corresponding author upon request.

Field-specific reporting

Please select the one below that is the best fit for your research. If you are not sure, read the appropriate sections before making your selection.

☒ Life sciences ☐ Behavioural & social sciences ☐ Ecological, evolutionary & environmental sciences

Life sciences study design

All studies must disclose on these points even when the disclosure is negative.

Sample size	Experiments were repeated a minimum of 3 times. Results were reproducible and statistical significance was apparent.
Data exclusions	No data were excluded from analyses.
Replication	Data were reproducible. Individual data points are shown throughout.
Randomization	Mice were randomized in experiments for gender and age between 6 weeks and 3 months.
Blinding	Investigators were not blinded during the course of experiments.

Reporting for specific materials, systems and methods

We require information from authors about some types of materials, experimental systems and methods used in many studies. Here, indicate whether each material, system or method listed is relevant to your study. If you are not sure if a list item applies to your research, read the appropriate section before selecting a response.

Materials & experimental systems

n/a	Involved in the study	
☐	☒	Antibodies
☒	☐	Eukaryotic cell lines
☒	☐	Palaeontology
☐	☒	Animals and other organisms
☒	☐	Human research participants
☒	☐	Clinical data

Methods

n/a	Involved in the study	
☒	☐	ChIP-seq
☐	☒	Flow cytometry
☒	☐	MRI-based neuroimaging

Antibodies

Antibodies used

Unlabeled anti-CD3 (eBioscience, clone 145-2C11; Invitrogen, clone OKT3), unlabeled anti-CD28 (1 µg/ml; eBioscience, clone 37.51; Invitrogen, clone CD28.2), APC-Cy7-conjugated anti-CD4 (BD Pharmingen, 552051, clone GK1.5), unlabeled anti-CD4 (R&D Systems, clone GK1.5), APC-conjugated anti-CD8α (BD Pharmingen, 557654, clone 53-6.7), unlabeled anti-CD8α (R&D Systems, clone 53-6.7), APC-Cy7-conjugated anti-CD4 (Biolegend, 300518, clone RPA-T4), PerCP-Cy5-5-A-conjugted anti-CD4 (BioLegend, clone OKT4), Alexa 700-conjugated CD8α (Biolegend, 344724, clone SK1), BV-605-conjugated anti-CD8 (BioLegend, clone RPA-T8), PerCP-Cy5.5-A-conjugated CD45.2 (Biolegend, 109828 , clone 104), Alexa Fluor 700A-conjugated CD45.1 (Biolegend, 110724, clone A20), V500-conjugated anti-CD4 (BD, 560782, clone RM4-5), APC-Cy7-conjugated anti-CD8α (BioLegend, 100714, clone 53-6.7), and PE-conjugated antiTCR Vβ5 (BD Pharmingen, 553190, clone MR9-4), unlabeled anti-LAMP-1 (eBioScience, clone 1D4B), unlabelled anti-LAMP-2 (Invitrogen, MA5-17861 clone M3/84) , Alexa 488-labeled donkey anti-rat (Thermo Fisher, A-21208), Alexa 594-labeled donkey anti-rat (Thermo Fisher, A-21209) PE-Cy7-conjugated anti-phospho S6 (Cell Signaling Technology, clone D57.2.2E), unlabeled anti-phospho NFkB p65 (Cell Signaling Technology, clone 93H1), Alexa 488-labeled donkey anti-rabbit (Jackson Immunoresearch), Alexa488-labeled donkey anti-rabbit (Jackson Immunoresearch, 711-545-152), unlabeled anti-phospho-S6 (Cell Signaling Technology, D57.2.2E), unlabeled anti-p44/p42 MAPK (T202/Y204) (Cell Signaling Technology, clone E10), unlabeled anti-phospho IκBα (S32/36) (Cell Signaling Technology, clone 5A5), unlabeled anti-MAPK (Cell Signaling Technology, clone 137F5), unlabeled anti-S6 (Cell Signaling Technology, clone 54D2), unlabeled anti-IκBα (Cell Signaling Technology, clone no. 9242).

Validation

Antibodies were characterized and validated for use by their respective manufacturers, by their use in published, peer-reviewed literature, and by appropriate experimental controls employed in each experiment.

Animals and other organisms

Policy information about studies involving animals; ARRIVE guidelines recommended for reporting animal research

Laboratory animals

Wild-type mice were bred in house and were on a mixed 129S6/SvEv X C57BL/6 genetic background. One exception was recipient mice in in vivo experiments that were on a CD45.1 C57BL/6 background (JAX). OTII TCR Tg mice (JAX) and Rasgrp1 mutant mice (JAX) were on a C57BL/6 genetic background. Mice ranged in age from 6 weeks to 3 months. Mice of both sexes were used in experiments. All experiments performed with mice were in compliance with University of Michigan guidelines and were approved by the University Committee on the Use and Care of Animals.

Wild animals

The study did not involve wild animals.

Field-collected samples | The study did not involve samples collected from the field.

Ethics oversight | All studies were approved by the University of Michigan Committee on the Use and Care of Animals.

Note that full information on the approval of the study protocol must also be provided in the manuscript.

Flow Cytometry

Plots

Confirm that:

☒ The axis labels state the marker and fluorochrome used (e.g. CD4-FITC).

☒ The axis scales are clearly visible. Include numbers along axes only for bottom left plot of group (a 'group' is an analysis of identical markers).

☒ All plots are contour plots with outliers or pseudocolor plots.

☒ A numerical value for number of cells or percentage (with statistics) is provided.

Methodology

Sample preparation	Murine spleens were ground with the flat end of a 1 mL syringe and passed through a 70 uM nylon mesh filter. Splenocytes were washed in PBS and erythrocytes were lysed by incubation in ACK lysis buffer for 5 minutes. Pan-T cells or CD4+ T cells were isolated from splenocytes by negative selection using the Miltenyi Biotec l Isolation kits and LS columns. Human PBMC were isolated from buffy coats by density centrifugation on Histopaque. After cell cuture, antibody staining of live or fixed cells was performed as described in the manuscript.
Instrument	All experiments described were performed on either a BD FACSCanto or BD Fortessa.
Software	Flow cytometry data were collected using FACSDiva v. 8.01 (BD) and analyzed in Flowjo v. 10.1 (Treestar).
Cell population abundance	All flow cytometry analyses were performed on samples containing a minimum of 10,000 CD4 and/or CD8 T cells as identified by the gating strategy outlined below.
Gating strategy	Viable cells were first gated on a plot of FSC-A vs. SSC-A. Doublets were then excluded by subsequent gating on FSC-H vs. FSC-A plots. Singlet CD4 and CD8-labeled cells were then gated on a bivariate plot of those markers. Labeled probe or intracellular phospho-S6 signal from within these subsets was then examined on histogram plots. For in vivo experiments, additional markers identifying other T cell subsets were incorporated into this scheme prior to probe or phospho-S6 measurements as described in the Methods.

☒ Tick this box to confirm that a figure exemplifying the gating strategy is provided in the Supplementary Information.
